# Establishment, immunological analysis, and drug prediction of a prognostic signature of ovarian cancer related to histone acetylation

**DOI:** 10.3389/fphar.2022.947252

**Published:** 2022-09-12

**Authors:** Yujie Fang, Jing Zhao, Xu Guo, Yunfeng Dai, Hao Zhang, Fanxin Yin, Xiaoxu Zhang, Chenxi Sun, Zequan Han, Hecheng Wang, Yanshuo Han

**Affiliations:** ^1^ School of Life and Pharmaceutical Sciences, Dalian University of Technology, Dalian, China; ^2^ Department of Radiotherapy, Yingkou Central Hospital, Yingkou, China; ^3^ Department of Pathology, Yingkou Fangda Hospital, Yingkou, China

**Keywords:** histone acetylation, ovarian cancer, prognostic markers, targeted therapy, immune therapy

## Abstract

In recent years, epigenetic modifications have been increasingly regarded as an important hallmark of cancer. Histone acetylation, as an important part of epigenetic modification, plays a key role in the progress, treatment, and prognosis of many cancers. In this study, based on the TCGA database, we performed LASSO regression and the Cox algorithm to establish a prognostic signature of ovarian cancer associated with histone acetylation modulator genes and verified it externally in the GEO database. Subsequently, we performed an immunological bioinformatics analysis of the model from multiple perspectives using the CIBERSORT algorithm, ESTIMATE algorithm, and TIDE algorithm to verify the accuracy of the model. Based on the prognostic model, we divided ovarian cancer patients into high-risk and low-risk groups, and assessed survival and the efficacy of accepting immunosuppressive therapy. In addition, based on the analysis of characteristics of the model, we also screened targeted drugs for high-risk patients and predicted potential drugs that inhibit platinum resistance through the connectivity map method. We ultimately constructed a histone acetylation modulator-related signature containing 10 histone acetylation modulators, among which HDAC1, HDAC10, and KAT7 can act as independent prognostic factors for ovarian cancer and are related to poor prognosis. In the analysis of the tumor microenvironment, the proportion of the B-infiltrating cells and the macrophages was significantly different between the high- and low-risk groups. Also, the samples with high-risk scores had higher tumor purity and lower immune scores. In terms of treatment, patients in the high-risk group who received immunotherapy had a higher likelihood of immune escape or rejection and were less likely to respond to platinum/paclitaxel therapy. Finally, we screened 20 potential drugs that could target the model for reference.

## Introduction

Ovarian cancer (OC) is one of the three major malignant tumors of the female reproductive system with the highest mortality rate (Siegel et al., 2020). Currently, more than 239,000 new cases of ovarian cancer occur worldwide each year (3.6% of all cancer cases), causing about 152,000 deaths each year (4.3% of all cancer deaths) (Reid et al., 2017). The convert location of ovarian cancer in the pelvic cavity accounts for the inconspicuous symptoms, and most patients are in the terminal stage when diagnosed due to the lack of effective screening methods. Also, the 5-year survival rate of patients with an advanced stage is only 29% (Lheureux et al., 2019). Tumor cell reduction and platinum-based chemotherapy are usually the initial treatment for ovarian cancer, but 70% of patients with epithelial ovarian cancer will relapse within 3 years (Ozga et al., 2015). And, multiple relapses lead to increasing resistance to chemotherapy drugs through a bewildering array of mechanisms (Holmes, 2015).

Studies have shown that the progression and treatment effect of OC are affected by many factors such as disease classification and staging, treatment strategy, and tumor microenvironment (Reid et al., 2017). Many transcriptional and epigenetic studies have also demonstrated that the occurrence, progression, and prognosis of OC are affected by the dynamic changes of multiple oncogenes and tumor suppressor genes (Ding et al., 2020). A few genes that may predict the prognosis of OC have been found in previous studies, but their clinical application is relatively limited.

Histone acetylation is a dynamically reversible process that determines the loose state of chromatin, and the relaxed chromatin in the acetylation state facilitates gene transcription, normally (Yang et al., 2018). The dynamic process of histone acetylation is controlled by a series of histone acetylation modulators (HAMs), which can be classified as Writers, Erasers, and Readers. Writers include histone acetyltransferases (HATs), which regulate gene transcription by adding acetyl groups to lysine residues of H3 or H4. Acetylation of histones is also removed by histone deacetylases (HDAC), a class of enzymes known as Erasers. In addition, proteins called histone acetylation readers recognize acetylated histones and recruit transcriptional mechanisms (Yun et al., 2011). These proteins generally contain bromodomain (BRD) or are themselves acetyllysine-binding proteins, such as the bromodomain and extra-terminal domain (BET) family. They are Readers that specifically bind acetylated histone H3/H4 and recruit downstream effectors to activate transcription (Jain and Barton, 2017). Readers identify lysine residues at the tail of acetylated histones by the BRD domain. This recognition is a prerequisite for protein–histone association and chromatin remodeling and is closely related to transcriptional activation (Filippakopoulos et al., 2012).

As an important part of epigenetic modification, histone acetylation plays an iconic role in the occurrence, development, and prognosis of many cancers. Unexpected high-frequency mutations in genes involved in the regulation of histone acetylation have been found in many cancers in recent genomic studies, suggesting that some HAMs may act as drive genes in cancer development (Hu et al., 2019). In epigenetic studies of breast cancer, samples with higher levels of acetylation of H4 showed a better prognosis and showed an overall decrease in the normal breast epithelium compared with the breast cancer tissue, suggesting that acetylation regulation has an impact on the prognosis of cancer (Elsheikh et al., 2009).

In recent years, targeted therapy and immunotherapy have become the key methods in the treatment of many advanced cancers due to their advantages of small toxicity and strong targeting (Topper et al., 2020). In terms of gene-targeted therapy, dysregulation of transcription due to altered protein acetylation patterns is a hallmark of cancer, and this is currently a mechanism by which HDAC inhibitors are targeted (Hrabeta et al., 2014). Presently, there are three HDAC inhibitors available for the clinical treatment of ovarian cancer; there are many targeted drugs for acetylation in preclinical trials, and more targeted drugs for histone acetylation are waiting to be discovered (Marsh et al., 2014). In terms of immunotherapy, many immune checkpoint inhibitor (ICI) drugs have been in the treatment of cancer. However, only a small number of patients can benefit from it due to the specificity of immunotherapy drugs. In addition, some cancers, such as pancreatic cancer, breast cancer, or ovarian cancer, seem to have intrinsic resistance to ICI drugs (Brahmer et al., 2012). How to identify the population that responds to immunotherapy drugs is a current problem.

In this study, we collated genes involved in acetylation regulation, established a novel prognostic signature associated with acetylation regulation using samples from TCGA and GTEx databases, and classified cancer patient populations into high-risk and low-risk groups. Based on CIBERSORT, ESTIMATE, and ssGSEA algorithms, we discussed differences in immune and survival characteristics among different risk subgroups in tumor immune infiltration. We focused on the expression of symbolic genes CA125 and HE4 and immune checkpoint genes PD-1, PD-L1, PD-L2, and CTLA4 in different groups of ovarian cancer so as to further explore the correlation between histone acetylation and the progress, treatment, and prognosis of ovarian cancer. To evaluate the role of risk subgroups in the treatment of ovarian cancer, IMVIgor210 and GSE30161 were introduced for validation. Furthermore, we used the MOA (mode of action) method in the cMAP database to screen out potential drugs targeting the model. In addition, gene functional enrichment analysis and protein interaction networks were used to improve the interaction mechanism of model genes. In conclusion, our study established a link between the expression of histone acetylation modulators and the progress, therapy, and prognosis of ovarian cancer, providing new ideas for the prognosis and treatment of ovarian cancer and providing help for ovarian cancer patients to find more effective targeted drugs.

## Materials and methods

### Data source

RNA-seq data of 375 serous ovarian cancer patients (the recurrence samples have been removed) and the corresponding clinical information were from the TCGA-OV data set (data version: 07-20-2019). RNA-seq data of 88 normal ovarian tissue samples were from the GTEx data set (data version:04-19-2016) (https://xenabrowser.net/datapages/). The expression microarray data and clinical information of 260 serous ovarian cancer patients from GSE32062 were downloaded from the GEO database (Yoshihara et al., 2012). The data format of the expression matrix adopts TPM (transcripts per kilobase million), and the standardized method is log_2_ (TPM+1). R package limma and sleuth were used for further quality control and data collation, and the average value of gene expression level was adopted for multi-probe genes.

A total of 77 acetylation regulatory genes were straightened out refer to the literature (Hu et al., 2019), including 22 histone acetylation genes (Writers), 18 histone deacetylation genes (Erasers), and 43 histone acetylation recognition genes (Readers), among which six genes serve as both Writers and Readers (Supplementary Table S1).

### Identification and enrichment analysis of differentially expressed histone acetylation modulators

Differentially expressed HAMs were analyzed by the limma package in R software (Friedman et al., 2010). To further investigate the biological functions of differential genes, Gene Ontology (GO) and Kyoto Encyclopedia of Genes and Genomes (KEGG) pathway enrichment analyses were conducted utilizing the GOplot package in R software.

### Construction and validation of the histone acetylation modulator-related signature for patients with ovary cancer

R package glmnet was applied to perform the LASSO regression algorithm to train the regression coefficient (Kamarudin et al., 2017). The optimal *λ* value was determined by 10-fold cross-validation and 1se analysis. Then the model genes were further screened by the Akaike information criterion (AIC), and a gene signature containing 10 HAMs was established by multivariate Cox regression. The risk score was obtained by the following formula:

Riskscore = Ʃ (Regression coefficients × Level of gene expression).

Therefore, TCGA ovarian cancer patients can be divided into high-risk and low-risk groups according to the median risk score.

To further verify the accuracy of the signature, a Kaplan–Meier curve was drawn by the survival package of R, the area under curve (AUC) of the time-dependent receiver operating characteristic (ROC) curve was analyzed by the time ROC package (Kamarudin et al., 2017), and a nomogram of overall survival (OS) prediction probability was established by the rms package as internal verification. Also, the GSE32062 data set acts as an external validation cohort. Risk scores of 260 OC samples were calculated using the abovementioned formula, and the KM curve and ROC curve were also used to verify the performance of the gene signature. Additionally, we reviewed previous literature on marker construction for ovarian cancer to ensure that AUC values had prognostic credibility (Liu et al., 2020a; Sun et al., 2019; Bao et al., 2020; Ding et al., 2020; Millstein et al., 2020).

### Correlation between gene expression and immune infiltration

Cibersort is a convolution tool for the expression matrix of immune cell subtypes based on linear support vector regression. The CIBERSORT source was downloaded (https://cibersort.stanford.edu/) and was then performed in the R platform. The whole-gene expression matrix of GTEx and TCGA was input to predict the relative proportion of 22 kinds of immune cells in the sample, and the expression of immune cells between the high-risk group and the low-risk group was compared. R package estimate was applied to predict the immune score and tumor purity of samples and to compare them in high- and low-risk groups.

In addition, we downloaded immune estimation data of TEGA samples from the TIMER database (Li et al., 2016) (https://cistrome.shinyapps.io/timer/). The relationship between the relative weight of immune cells (CD4^+^ T-cells, CD8^+^ T-cells, B-cells, and macrophages) and risk score was explored, and the correlation chart was drawn.

### Therapeutic effect evaluation

In order to evaluate the response of different subgroups of patients to ICIs, we first compared the expression levels of four important immune checkpoint genes. Furthermore, the TIDE algorithm was used to evaluate the possibility of each patient’s tumor immune escape (Fu et al., 2020) (http://tide.dfci.harvard.edu/).

IMVIgor210 was the immunotherapy cohort introduced as an external validation cohort to verify the consistency of the immunotherapy effect and prediction (Zhang et al., 2020a). GSE30161 was used to study the relationship between the model and platinum resistance, which contains 58 patients with ovarian cancer receiving platinum chemotherapy (Ferriss et al., 2012).

### Mutation analysis

The mutation profile of OC samples was derived from the TCGA database. The R package maftools is used to process and analyze data in MAF format. We visualized the frequency of mutations in the high/low-risk groups and calculated the tumor mutation burden (TMB) score for each sample: TMB = (total mutations/total covered bases) ×10^6.

### Prediction of potential target compounds for ovarian cancer patients

We utilized Broad’s CMap database to predict potential drugs that target the HAM-related signature (Subramanian et al., 2017). The mode of action (MoA) analysis was used to sort out the class and mechanism of drugs.

### Identification of crucial prognostic histone acetylation modulators

Survival analysis was used to explore the prognostic value of signature genes and to identify genes with independent prognostic ability. Subsequently, protein expression of these independent prognostic factors was confirmed in the Human Protein Atlas (HPA) database (http://www.proteinatlas.org/) (Thul et al., 2017).

### Protein expression analysis

The STRING database and the geneMania database were used to build the Protein–protein interaction (PPI) network. The STRING database (https://string-db.org) depicts a network of physical and functional interactions of proteins based on systematic co-expression analysis and literature text mining (Szklarczyk et al., 2017). PPI network analysis was then constructed to predict physical and functional interactions of prognostic HAMs to explore the core genes of the network. The GeneMANIA database (http://www.genemania.org) can be used to identify genes associated with signatures for further analysis (Franz et al., 2018). Interaction networks associated with signature genes were constructed by identifying gene co-annotation patterns in gene ontology or using enrichment analysis.

### Statistical analysis

In this study, all statistical analyses were conducted using Perl software (version 5.32.1.1) and R software (version 4.1.1). Wilcoxon test and Kolmogorov–Smirnov test were used to compare paired groups. In addition, *p* value < 0.05 was considered as statistically significant.

## Result

### Differential expression analysis of histone acetylation modulators in OV

**FIGURE 1:**
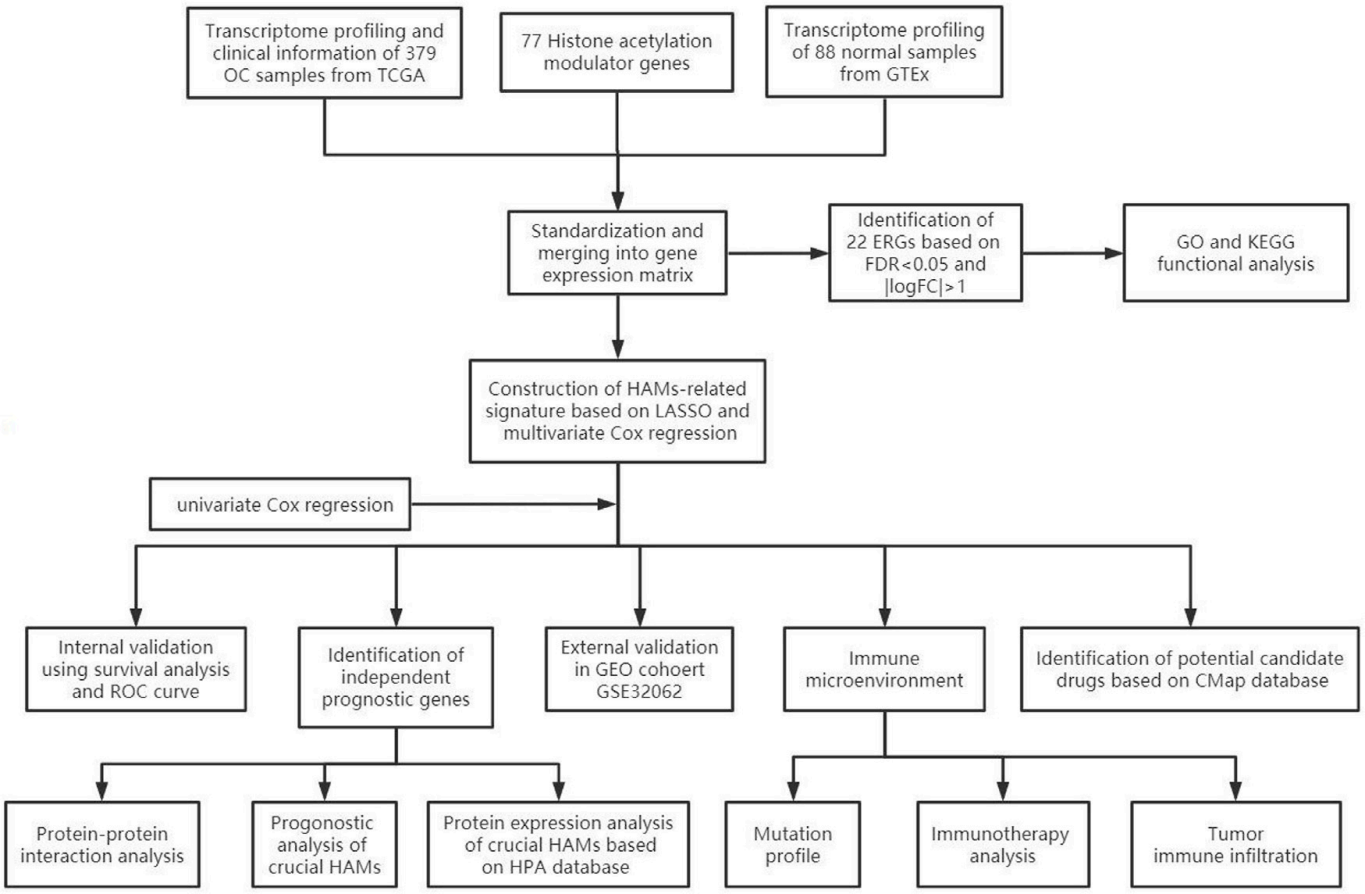
Flow diagram of this study.

**FIGURE 2:**
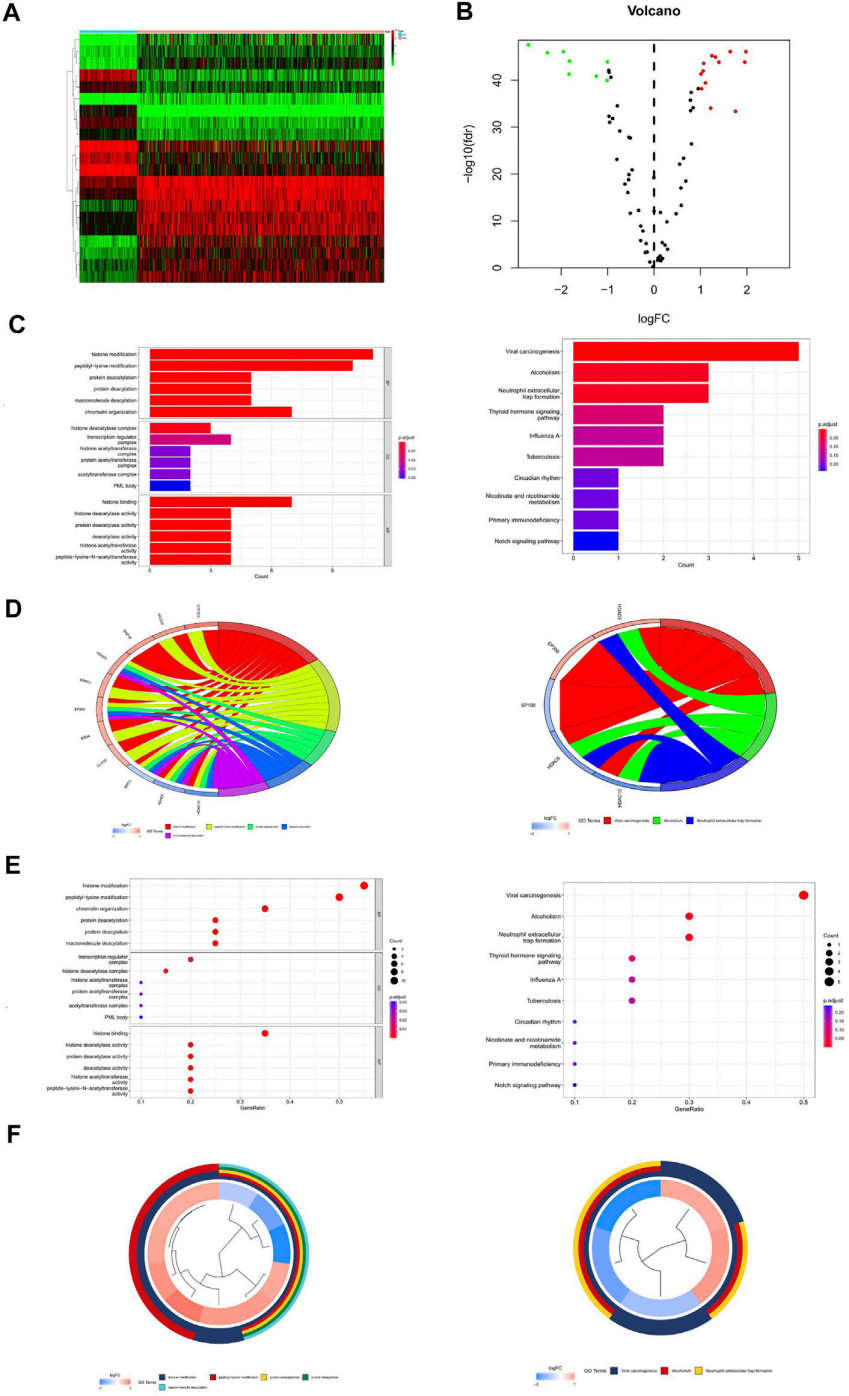
Differential expression analysis and gene enrichment analysis. **(A)** Heat map of differentially expressed genes. **(B)** Volcano map of differentially expressed genes. **(C)** Enrichment analysis histogram of GO and KEGG analysis. **(D)** Enrichment analysis bubble plot of GO and KEGG analysis. **(E,F)** Enrichment analysis circle diagram of GO and KEGG analysis.

The workflow of our study is illustrated in Figure 1. First, we downloaded mRNA data of 375 OV samples and 88 corresponding normal ovary samples with clinical information from the TCGA and GTEx databases. By comparing the gene expression profiles in the TCGA cancer group and the GTEx normal group, a total of 21 differentially expressed genes (DEGs) were identified among 77 HAMs genes (|logFC|>1,*p* < 0.001), with eight downregulated genes and 13 upregulated genes(Figure 2A,2B). Compared with the normal group, the expression of SP110 and HDAC10 reached −2.70 and −2.30 logFC, respectively, which were significantly downregulated. In contrast, the expression of ATAD2 and ZMYND8 were upregulated with logFC of 1.96 and 1.98, respectively. All differential expression results are presented in Supplementary Table S2.

### Gene Ontology and Kyoto Encyclopedia of Genes and Genomes functional analysis

Through GO enrichment analysis, the differentially expressed genes were mainly enriched to 77 items (q-value<0.05), including histone modification, peptidyl-lysine modification, histone binding, histone deacetylase activity, and so on (Supplementary Table S3). The results of KEGG analysis showed that HAM differential genes were mainly enriched in three pathways (q-value<0.05): viral carcinogenesis, alcoholism, and neutrophil extracellular trap formation (Supplementary Table S4) (Figures 2C–F).

### Development of the histone acetylation modulator-related signature

The TCGA cohort (TCGA-OV, *n* = 375) is used as a training set to construct HAM-related signatures. To eliminate the overfitting of gene signature, LASSO regression was performed to screen genes and train regression coefficients. Then, genes that contributed less to the model were filtered out by the AIC criterion. Finally, a 10-gene prognostic marker was obtained, and the formula was as follows:

Riskscore=(-0.3818)*ELP3+(0.2097)*HDAC1+(0.2688)*HDAC10+(0.2029)*HDAC11+(0.1501)*HDAC2+(0.3085)*HDAC4+(0.4347)*KAT7+(-0.3345)*KIAA 2026+(-0.4711)*SIRT5+(-0.1796)*SP140.

**FIGURE 3:**
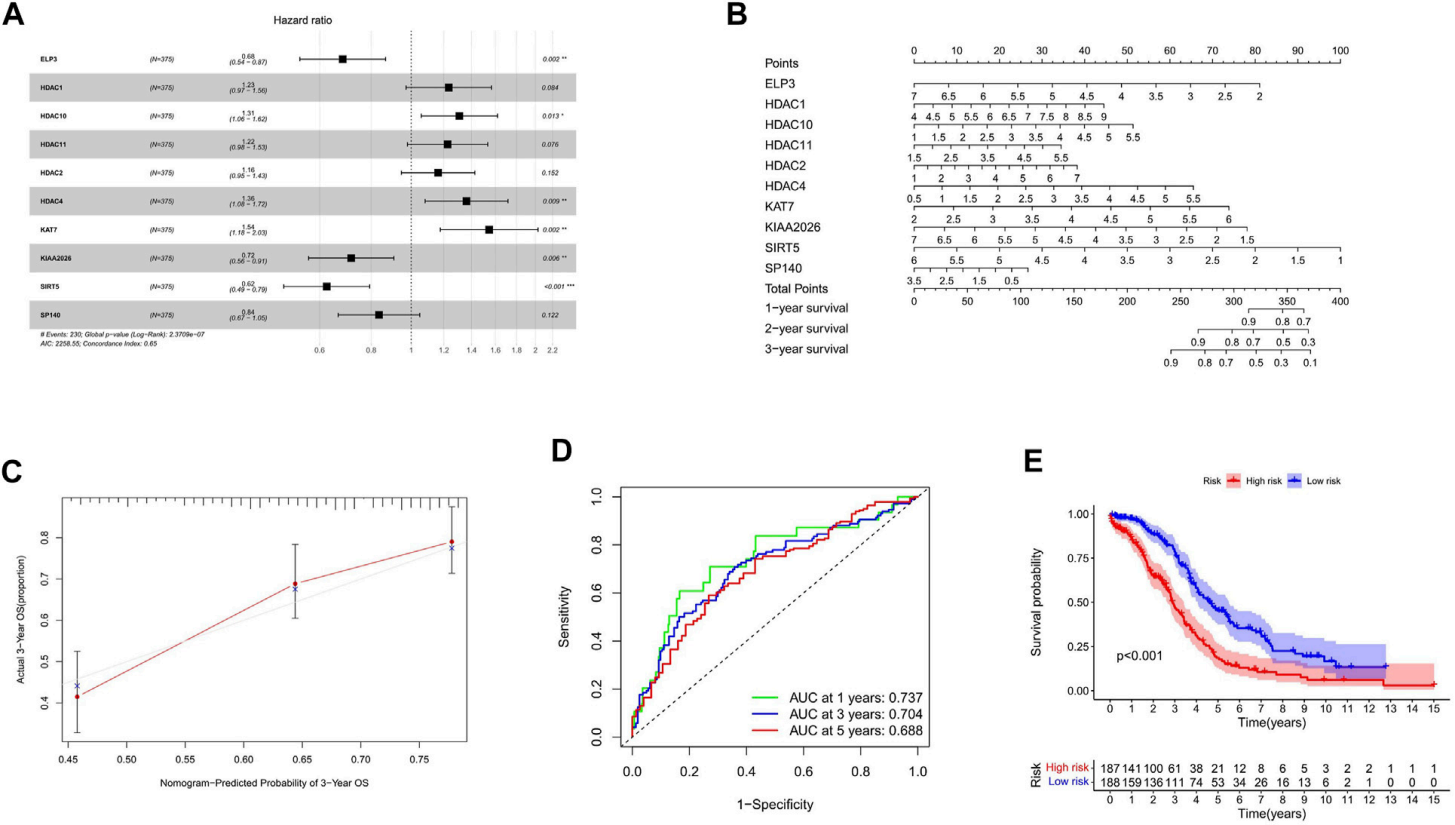
Construction of HAM signature by LASSO regression. **(A,B)** Prediction of immune cell proportion in TCGA and GTEx samples. **(C)** Calibration curve of 3-year OS. **(D)** Time-dependent ROC curves predicted 1-, 3-, and 5-year prognostic performance in the training cohort. **(E)** Kaplan–Meier curves to compare the OS of high‐risk and low‐risk groups in a training cohort.

According to the calculated median Risk score, TCGA patients were then divided into high-risk and low-risk groups (Supplementary Table S5). The hazard ratio of the signature was presented (Figure 3A). As shown in Figure 3A, HDAC1, HDAC2, HDAC4, HDAC10, HDAC11, and KAT7 genes in the HDAC family were bad prognostic factors (hazard ratio>1), while ELP3, KIAA 2026, SP140, and SIRT5 were good prognostic factors (hazard ratio< 1). Furthermore, the line diagram and calibration diagram of the model were also performed (Figures 3B,C).

The time-dependent ROC curve analysis and Kaplan–Meier curves were performed (Figures 3D,E) for internal validation of the model. The AUC values of HAM-related signatures at 1, 3, and 5 years were 0.737, 0.704, and 0.688, respectively. In order to achieve a horizontal comparison, a table that includes several previous modeling of prognostic signatures for ovarian cancer was compiled (Supplementary Table S6).

### External validation of the histone acetylation modulator-related signature

**FIGURE 4:**
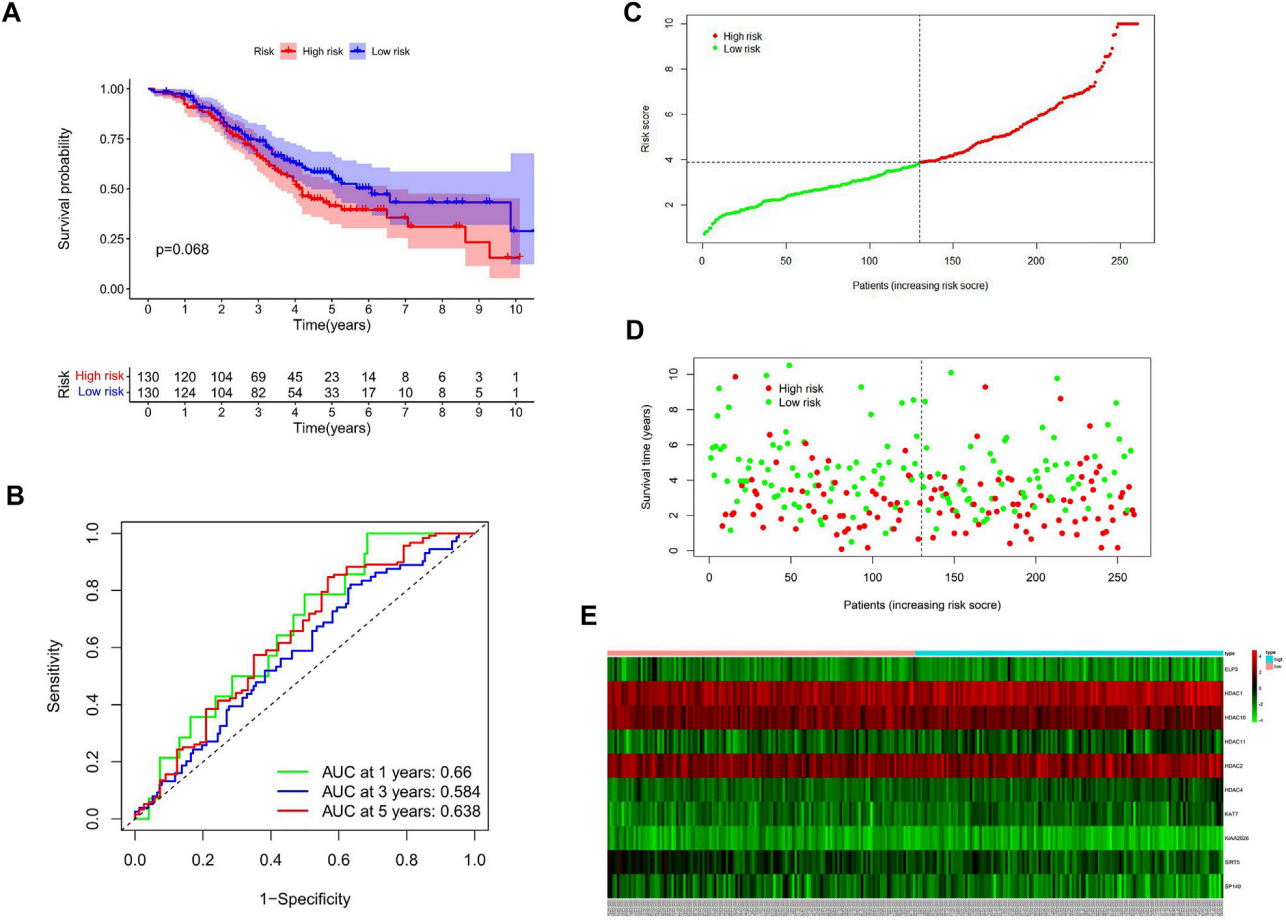
Validation of HAM signature in the GEO cohort. **(A)** Kaplan–Meier curves to compare OS of high‐risk and low‐risk groups in the validation cohort. **(B)** Time-dependent ROC curves predicted 1-, 3-, and 5-year prognostic performance in the validation cohort. **(C)** Risk score distribution. **(D)** Individual status of survival. **(E)** Heat map of the differentially expressed genes between high and low risks.

GSE32062 (*n* = 260) is a large ovarian cancer data set from the GEO database, which is used for external validation of the gene signature. Consistent with internal validation, the Kaplan–Meier curves (Figure 4A) showed that the high-risk group had a poorer prognosis. The time-dependent ROC curve analysis was conducted, and the AUC values were 0.66, 0.584, and 0.638 of 1, 3, and 5 years of prognostication, respectively (Figure 4B).

### Comparing the immune infiltration between the subgroups

**FIGURE 5:**
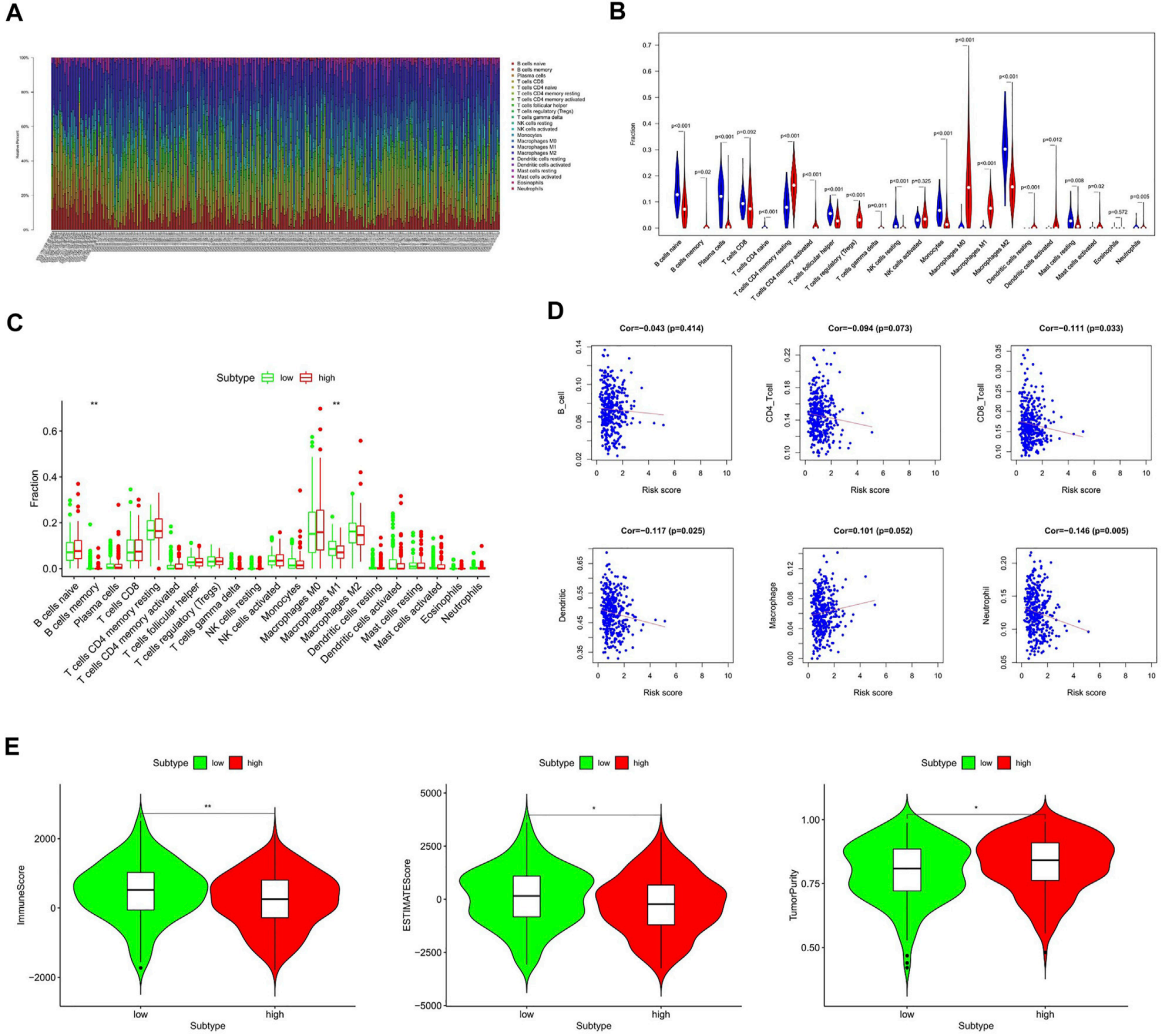
Relationship between the HAM signature and immune infiltration. **(A,B)** Prediction of immune cell proportion in TCGA and GTEx samples. **(C)** Comparison of relative immune cell abundance in high-risk and low-risk groups. **(D)** Correlation of risk score and immune infiltrates. **(E)** Comparison of tumor immune score in high-risk and low-risk groups based on the ESTIMATE R package. *Statistically significant *p* < 0.05; **: statistically significant *p* < 0.01.

Tumor immune infiltration is one of the main biological characteristics of various cancers and is significantly related to the prognosis. To study the inner relationship between immune infiltration and the gene signature, we predict the distribution of 22 immune cells in the TCGA and GTEx cohorts by the CIBERSORT algorithm at first. The heat map and violin diagram (Figures 5A,B) indicate that the distribution of macrophages, B-cells, and CD4 cells have significant differences between tumor tissues and normal tissues.

Next, we compared the immune infiltration of TCGA samples in the high- and low-risk groups (Figure 5C). It shows that the B-memory cells and macrophage M1 were significantly different between high- and low-risk groups, indicating a correlation with prognosis. Furthermore, the relationship between six immune cells and the risk score was comprehensively compared (Figure 5D). Thereinto, the infiltration levels of CD8 cells, dendritic cells, and neutrophils were negatively correlated with the risk score, while the macrophages were significantly correlated with the higher risk.

Subsequently, we applied the ESTIMATE algorithm to calculate the immune score, estimate score, and tumor purity between low-risk and high-risk groups (Figure 5E). As shown in Figure 5E, patients with high-risk scores had significantly lower immune scores than those in the low-risk group, while tumor purity was significantly higher compared to the low-risk group.

### Evaluation of immune status between the subgroups

**FIGURE 6:**
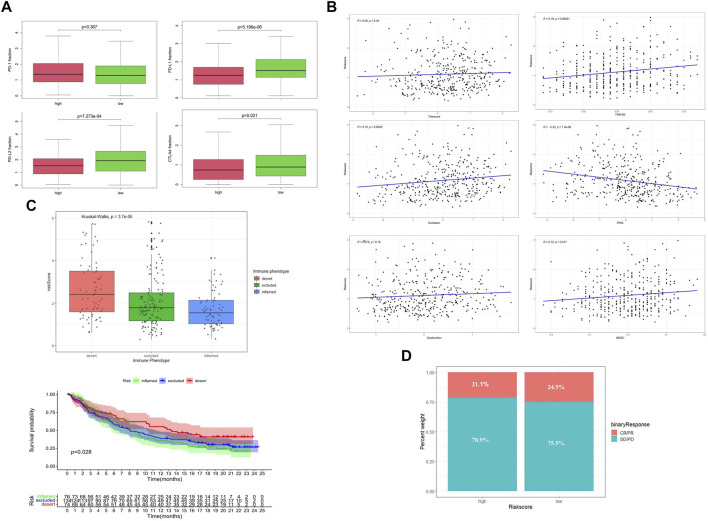
Evaluation of immune status between low-risk and high-risk groups. **(A)** Expression level of immune checkpoint genes. **(B)** Tumor immune dysfunction and exclusion scores in the high- and low-risk groups. **(C)** Relationship between risk score and the immune phenotype in the IMVIgor210 cohort. **(D)** Objective response rates in the low-risk group (ORR = CR + PR).

By comparing the gene expression profiles of TCGA patients, we found that the expressions of CTLA4, PD-L1, and PD-L2 were significantly different among different risk groups, and the expression levels were higher in the low-risk group (Figure 6A). Applying the TIDE algorithm, we calculated the Tidesore, Exclusion score, and Dysfunction score of each sample, and the scores of all three were high in the high-risk group. In addition, the infiltrating results of myeloid-derived suppressor cells (MDSCs) and the M2 subtype of tumor-associated macrophages (TAM.M2) and the expression of interferon-γ (IFNG) were significantly different between the different risk subgroups (Supplementary Table S3) and correlated with the risk score (Figure 6B).

To further explore the role of the risk score model in predicting the immune response of patients, we introduced the IMVIgor210 cohort for analysis (Supplementary Table S7). With the increase of risk score, the immune state of patients changed from inflamed to excluded and desert (Figure 6C), and the degree of immune infiltration decreased periodically. In addition, patients with high-risk scores had a lower objective response rate (ORR) to ICI than those in the low-risk group. (Figure 6D).

### Mutation profile and histone acetylation modulator risk groups

**FIGURE 7:**
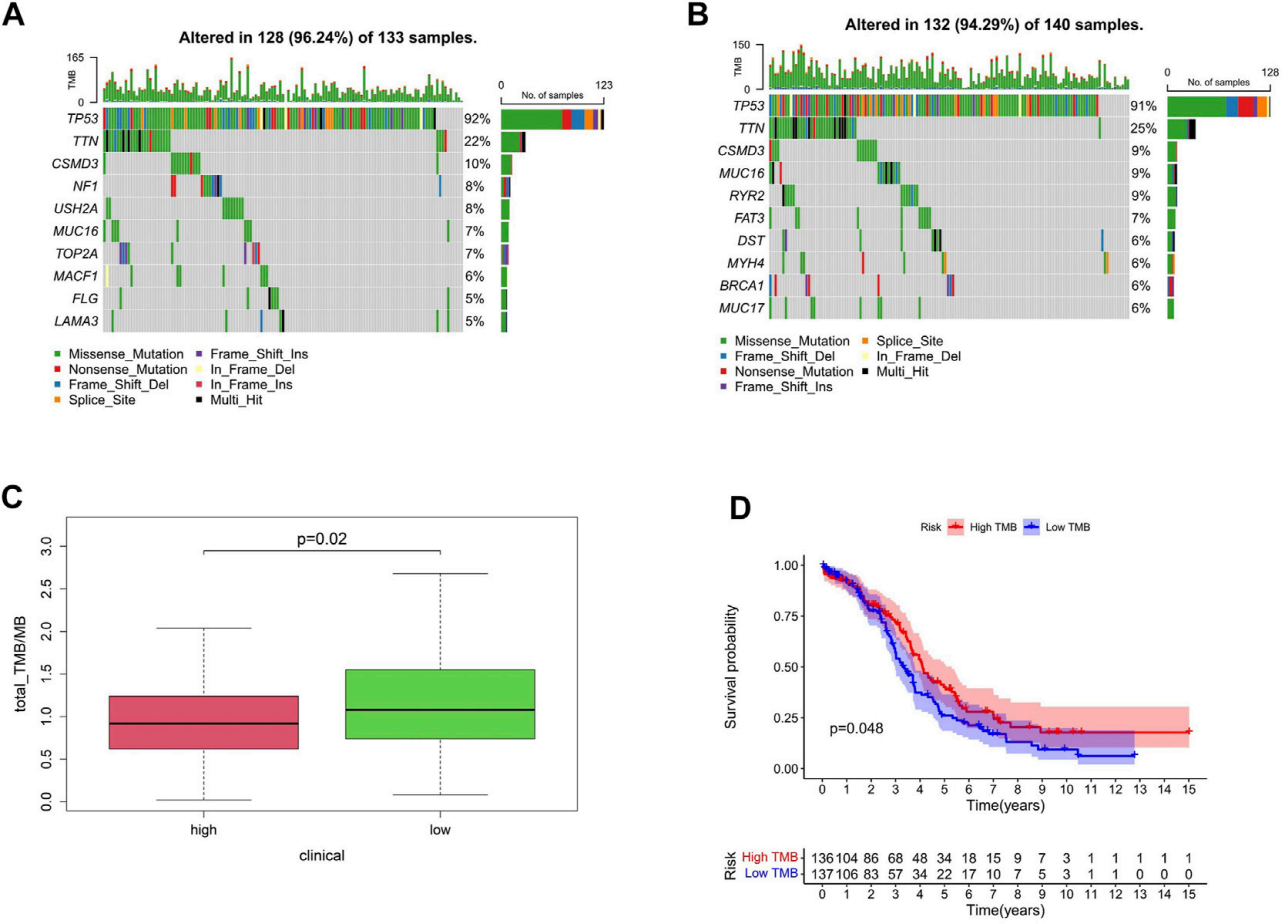
Mutation profile and HAM risk groups. **(A,B)** Mutation profiles of high- and low-risk groups. **(C)** TMB differences between high- and low-risk groups. **(D)** Correlation between TMB and survival.

Gene mutation is one of the main reasons for tumor occurrence and progress. By evaluating the frequency of tumor mutation, the tumor mutation burden (TMB) of patients can be calculated. According to the model established, the proportion order of somatic mutations in high-risk group was TP53 > TTN > CSMD3 > NF1 > USH2A > MUC16 (CA125) >TOP2A > MACF1>FLG > LAM (Figure 7A) and that in the low-risk group was TP53 > TTN > CSMD3 > MUC16 (CA125) > RYR2 > FAT3 > DST > MYH4 > BRCA1 > MUC17 (Figure 7B). TMB is an important indicator currently used to evaluate immunotherapy, clinically. Compared with patients with a high-risk score, TMB was significantly increased in the low-risk group, and TMB was correlated with patient survival (Figures 7C,D).

### Targeted therapy analysis and drug prediction

**FIGURE 8:**
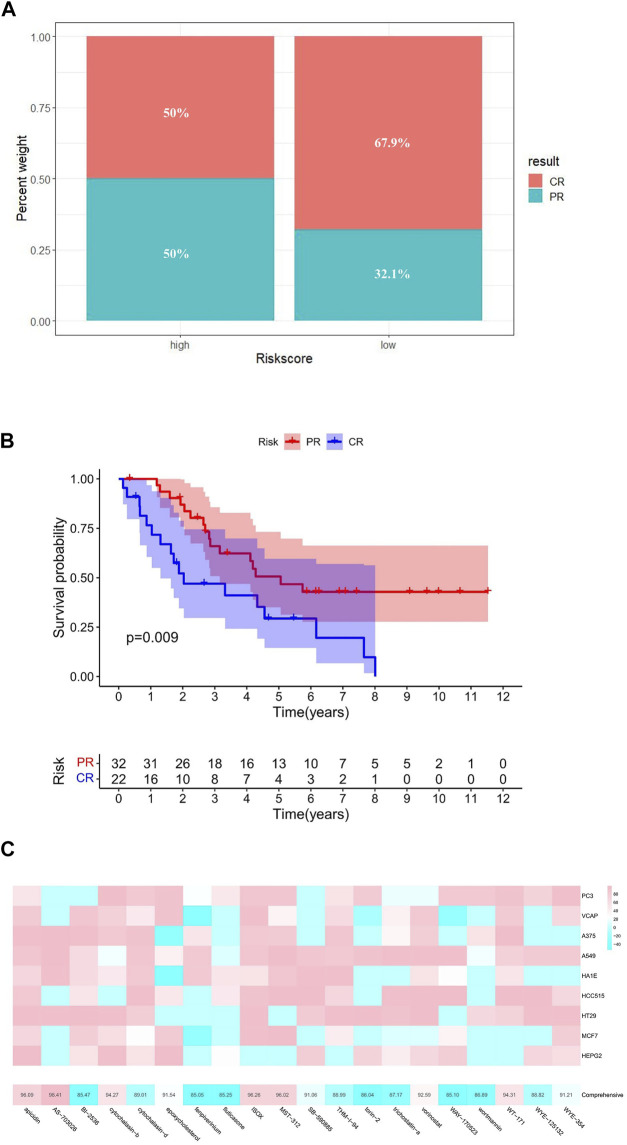
Targeted therapy analysis. **(A)** Efficacy of platinum/paclitaxel in the high- and low-risk groups. **(B)** Relationship between therapeutic effect and survival. **(C)** Potential targeted drugs predicted by cMAP analysis (the abscissa of the heatmap is the compound and score, the ordinate is the cell line, red indicates sensitivity to the compound, and blue indicates insensitivity to the compound).

Subsequently, based on the GSE30161 data set, we compared the prognosis of patients receiving platinum/paclitaxel with different levels of risk scores (Supplementary Table S8). The result showed that the high-risk group had a lower percentage of complete response (CR) after treatment (Figures 8A,B).

By comparing gene expression characteristics between patients in the high- and low-risk groups (Supplementary Table S9), we used MoA analysis of CMap to identify 20 ideal compounds for targeting genetic signatures (Figure 8C). The mechanisms of these drugs include HDAC inhibitor, CDK inhibitor, PLK inhibitor, mTOR inhibitor, MEK inhibitor, and so on. Among them, HDAC inhibitor serves as the main pathway toward the signature, consisting of six drugs: ISOX (score = 96.26), APicidin (score = 96.09), WT-171 (score = 94.31), Vorinostat (score = 92.59), THM-I-94 (score = 88.99), and Trichostatin-a (score = 87.17) (Table 1).

**TABLE 1:** Scores and mechanisms of 20 potential drugs.

Name	Comprehensive Score	MOA (mechanism of action)
AS-703026	98.41	MEK inhibitor
ISOX	96.26	HDAC inhibitor
apicidin	96.09	HDAC inhibitor
MST-312	96.02	telomerase inhibitor
WT-171	94.31	HDAC inhibitor
cytochalasin-b	94.27	microtubule inhibitor, phagocytosis inhibitor
vorinostat	92.59	HDAC inhibitor, cell cycle inhibitor
epoxycholesterol	91.54	LXR agonist
WYE-354	91.21	mTOR inhibitor
SB-590885	91.06	RAF inhibitor
cytochalasin-d	89.01	Actin polymerization inhibitor, actin stabilizer
THM-I-94	88.99	HDAC inhibitor, apoptosis stimulant, cell cycle inhibitor
WYE-125132	88.82	mTOR inhibitor, PI3K inhibitor
trichostatin-a	87.17	HDAC inhibitor, CDK expression enhancer, ID1 expression inhibitor
wortmannin	86.89	PI3K inhibitor, ATM kinase inhibitor, PLK inhibitor, etc.
torin-2	86.04	mTOR inhibitor
BI-2536	85.47	PLK inhibitor, apoptosis stimulant, cell cycle inhibitor, protein kinase inhibitor
fluticasone	85.25	Glucocorticoid receptor agonist
WAY-170523	85.1	Metalloproteinase inhibitor
fenpiverinium	85.05	Acetylcholine receptor antagonist

### Exploring crucial independent prognostic histone acetylation modulators

**FIGURE 9:**
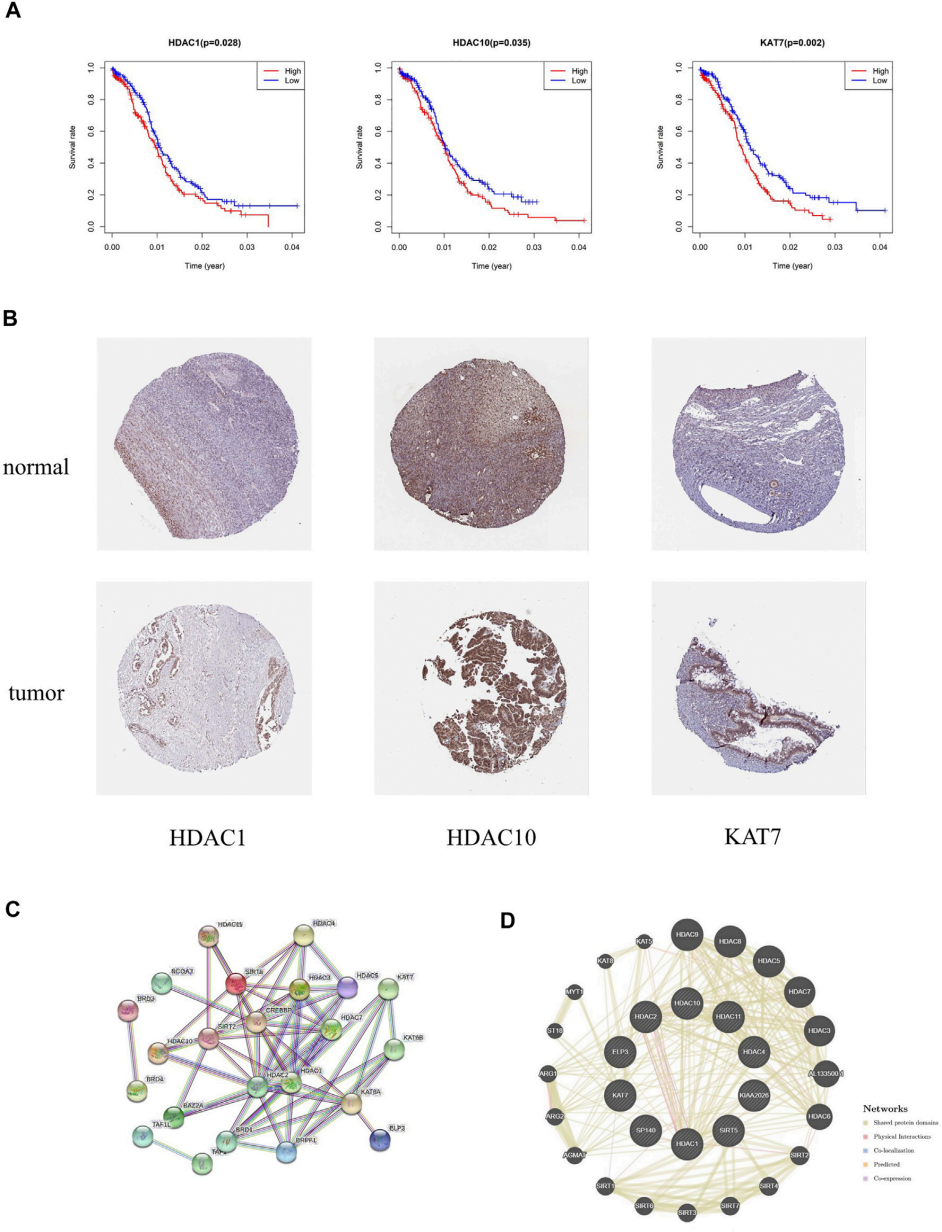
**(A)** Kaplan–Meier survival curves revealed contrasting survival possibilities predicted by varying expression of signature genes. **(B)** Protein expression of crucial HAMs in ovary cystadenocarcinoma serous and normal ovary tissues based on the HPA database (HDAC1 tumor: HPA029693, staining: medium, quantity: >75%; HDAC1 normal: CAB068191, staining: medium, quantity: >75–25%; HDAC10 tumor: CAB045977, staining: medium, quantity: >75%; HDAC10 normal: CAB045977, staining: high, quantity: >75%; KAT7 tumor: HPA044470, staining: medium, quantity: 75–25%; KAT7 normal: HPA044470, staining: medium, quantity: >75–25%). **(C,D)** PPI network of prognostic HAMs. **(C)** PPI network by the STRING database. **(D)** Protein interaction analysis by the geneMania database.

Survival analysis was applied to identify the prognostic value of HAM signature genes in the TCGA cohort. Only HDAC1 (*p* = 0.028), HDAC10 (*p* = 0.035), and KAT7 (*p* = 0.002) were tested for significant survival correlations (Figure 9A). In addition, lower expression of HDAC1, HDAC10, and KAT7 resulted in relatively longer survival, while SP140 (*p* = 0.064) was detrimental to survival (Supplementary Table S4).

### Protein expression analysis of crucial histone acetylation modulators

The immunohistochemical diagram of the HPA database is presented in Figure 9B. The results showed that the protein expression level of HDAC10 was significantly downregulated in tumor samples. Moreover, the protein expression levels of HDAC1 and KAT7 were also downregulated to a certain extent, which was consistent with the results of the survival analysis.

Based on the STRING and geneMania databases, we have explored the protein interaction network of the signature genes (Figures 9C,D). The STRING database shows the inner interaction network. The result indicates that CREBBP, HDAC1, and HDAC2 possess the most internal interactions. Furthermore, through protein interaction analysis conducted by the geneMania database, we found some genes associated with HAM signature, such as HDAC9, HDAC8, ALL133500.1, HDAC6, AGMAT, and so on.

## Discussion

Ovarian cancer is one of the diseases with the highest mortality rate of gynecological malignant tumors (Stewart et al., 2019). The lack of effective and sensitive diagnosis means that in the early stage of ovarian cancer, the cancer has chemotherapy resistance and metastasis in the advanced stage, resulting in poor treatment effect and prognosis of patients (Lisio et al., 2019). Therefore, rapid and accurate early diagnosis and rational medication and treatment strategies are the key to the treatment of ovarian cancer. Genetics and epigenetics are two key factors that determine the occurrence and development of tumors. A large number of epigenetic modification-related genes are changed at a high frequency in cancer and may become driving genes in the process of cancer development (Hu et al., 2019). Histone acetylation, involved in the regulation of the cell cycle, cell differentiation, and apoptosis, greatly affects the occurrence, development, and treatment of cancer (Farria et al., 2015).

In this study, we studied 77 important HAMs, including Writer, Eraser, and Reader. Through GO and KEGG enrichment analysis, these HAMs are mainly involved in the modification of histones and the regulation of a variety of transcriptional activities, thus playing an important role in the progress, development, and prognosis of cancer. By analyzing the transcriptional expression profile of HAMs of ovarian cancer patients in TCGA and normal samples in GTEx, we established a prognostic signature of ovarian cancer associated with HAM genes and verified it in the GEO database. According to the risk score of our model, patients can be divided into high-risk and low-risk subgroups, and the OS of patients in the two subgroups is significantly different in both the training cohort and the validation cohort. The results of the ROC curve and nomogram indicate that the risk model established by us is effective in prognosis.

Among the multi-gene signature established by us, there are 10 HAMs, among which two belong to the histone acetylation enzyme (ELP3, KAT7), two belong to the acetylation reader (SP140, KIAA 2026), and the other six belong to the histone deacetylation enzyme (HDAC1, HDAC2, HDAC4, HDAC10, HDAC11, and SIRT5). KAT7 (HBO1) belongs to the MYST superfamily and contains a specific region composed of the acetyl-CoA binding motif and zinc finger (MYST domain). ELP3 belongs to the GNAT superfamily and has a conserved GNAT domain and can acetylate lysine residues on histone H3 (Srivastava et al., 2014; Salah et al., 2016); HDAC1 and HDAC2 are Class I HDAC, which are nuclear proteins. HDAC4 and HDAC10 are Class II HDACs, which travel between the cytoplasm and nucleus. HDAC11 is a Class IV HDAC with shared properties of Class I and CLASS II. It is an NAD + dependent enzyme. Figure 3A shows that among the six HDACs, only SIRT5 is a benign prognostic factor, while the rest are associated with a poor prognosis of ovarian cancer.

In previous studies, these HAM proteins have been demonstrated to be closely associated with the progress of many cancer, and different types of HAMs have different effects on cancer. The catalytic subunit of the histone acetyltransferase KAT7 complex mediates the acetylation of histone H3K14ac, H4K5ac, H4K8ac, and H4K12ac, thus playing a regulatory role in gene transcription, protein ubiquitination, and immune regulation (Iizuka and Stillman, 1999; Doyon et al., 2006). Studies have shown that KAT7 enhances the mechanical transduction pathway and membrane elasticity of ovarian cancer cells through the overexpression of preferential acetylation histone H4 of co-mediator JADE2, thus improving the migration ability and invasiveness of ovarian cancer cells (Quintela et al., 2019; Gao et al., 2021). SP140 acts as an acetylation reader, preferentially occupying promoters of silenced genes with histone modification of H3K27me3, and is critical for transcriptional programs that support the macrophage state (Mehta et al., 2017). Histone deacetylases HDACs are the most important components of the gene labels we have established. Among them, HDAC1 and HDAC2 are class I HDACs, whose increased expression is an independent risk factor for poor prognosis of malignant ovarian tumors (Khabele et al., 2007; Weichert et al., 2008). In ovarian cancer, HDAC1 promotes cancer cell proliferation by increasing cyclin A (Hayashi et al., 2010), and HDAC2 interferes with cisplatin-induced activation of DNA damage responses by remodeling chromatin (Huang et al., 2016). In addition, many studies have shown that HDAC1 is also a good diagnostic or prognostic signature for lung cancer, gastric cancer, glioma, breast cancer, and other cancers (Cao et al., 2017; Yu et al., 2019; Guo et al., 2020; Yang et al., 2020). HDAC4 and HDAC10 are Class II HDACs, which are associated with proliferation, migration, and invasion of a variety of cancers (Cai et al., 2018; Cheng et al., 2021). SIRT5 is a Class III HDAC that is involved in oxidative stress or metabolic homeostasis related to aging, degeneration, or cancer (Gonzalez-Fernandez et al., 2019). Relevant studies revealed that SIRT5 can promote autophagy of gastric cancer cells, and SIRT5 can inhibit peroxisome-induced oxidative stress, thus protecting the liver and inhibiting the development of hepatoma cells (Chen et al., 2018; Gu et al., 2021). HDAC11 is the most recently discovered and the smallest member of the HDAC enzyme. At present, HDAC11 has been found to be associated with poor prognosis in the liver, lung, ovarian, glioma, uveal melanoma, and other cancers (Yanginlar and Logie, 2018; ; Liu et al., 2020b; Bi et al., 2021). The results of the current literature are consistent with our findings, further confirming the widespread role of HAMs in cancer and supporting the prognostic value of these genes for ovarian cancer and other cancers.

We focused on the relationship between the risk signature and the tumor microenvironment. According to the CIBERSORT algorithm analysis, 19 of the 22 types of immune cells had significant differences between tumor samples and normal samples. Among them, the expression of macrophages M0, macrophages M1, Tregs, and CD4^+^ T-cells were significantly upregulated in cancer, indicating that they are important factors involved in ovarian cancer immunity. However, the infiltration of B-cells and macrophage M1 showed significant differences between the samples in the high- and low-risk groups. Furthermore, correlation analysis showed a negative correlation between the degree of B-cell infiltration and the risk score, suggesting a potential tumor suppressive effect of enhanced B-cell infiltration. Recent studies have also shown that tumor-infiltrating B-cells have antitumor effects and can combine with CD4^+^ T-cells to enhance local immune responses (Zhang et al., 2020b). Subsequently, we performed the ESTIMATE algorithm to assess the overall immune status of ovarian cancer patients. Among them, the tumor purity was higher in the high-risk group, while the immune score was significantly reduced. This indicates that patients with a high-risk score had a poor level of tumor immune infiltration and were less able to kill tumor cells. Meanwhile, the result revealed a negative correlation between the risk score of the model and the level of immune infiltration.

In studies of immunotherapy efficacy, we examined the expression levels of several important immune checkpoint genes. We found that the expression of PD-L1, PD-L2, and CTLA4 was significantly increased in the low-risk group, and it can be speculated that the low-risk patients might have more obvious effects after receiving immune checkpoint inhibitors. In addition, the TIDE algorithm revealed a higher likelihood of immune escape or immune dysfunction in high-risk patients, heralding poor response to immunoblocking therapy (ICB) in these patients. In addition, mutation analysis showed a significant decrease in TMB in the high-risk group compared to the low-risk group, and this difference may have an impact on patient survival. To further verify the effect of receiving immunotherapy, we introduced the IMVIgor210 cohort treated with PD-1/PD-L1 inhibitors as the validation set. The results indicated that patients in the high-risk group had a lower, but not significant, rate of objective response (CR/PR) after ICI treatment. Patients tended to perform differently on the immune phenotype according to different risk scores. However, with the increase of risk score, the immune phenotype of patients varies from “inflamed” to “exclude” and then to “desert,” indicating a decline in the level of immune infiltration and the effect of receiving immunotherapy (Chen and Mellman, 2017).

Our study also provides potential drugs for target therapies. We have identified 20 compounds targeting HAM-related signature using cMAP as potential target drugs for OC patients. These drugs include HDAC inhibitor, CDK inhibitor, PLK inhibitor, mTOR inhibitor, MEK inhibitor, and so on. Of concern, six of the 20 small molecule compounds we identified act as histone deacetylation inhibitors (HDACIs). HDACIs are a diverse group of compounds that vary in structure, bioactivity, and specificity. By affecting transcription, HDACIs can halt the cell cycle, inhibit DNA repair, and induce apoptosis and acetylation of non-histones, leading to downstream changes in gene expression (Lakshmaiah et al., 2014). On one hand, dysregulation of transcription due to altered histone acetylation patterns is a mechanism for cancer occurrence, which is currently targeted by HDAC inhibitors; on the other hand, the traditional treatment for ovarian cancer is generally platinum-based therapy, while the high expression of HDAC family members increases the resistance of patients to platinum chemotherapy. Islam et al. (2017) have identified HDAC10 inhibitors as potential therapeutic targets for ovarian cancer, enhancing the efficacy of platinum drugs in malignant ovarian tumors. In addition, studies have shown that silencing HDAC1 by siRNA targeting leads to the induction of xenograft tumors that are sensitive to cisplatin therapy and can reduce drug resistance, which may be an effective strategy to improve the efficacy of cisplatin therapy (Liu et al., 2018). In the sample analysis of GSE30161, our results verified this: high-risk patients had a significantly lower CR ratio compared with low-risk patients due to higher HDAC expression. Therefore, the combination of HDAC inhibitors and platinum drugs may become one of the effective strategies for the treatment of ovarian cancer.

In this study, multiple data sets were included in the model construction and validation process to improve its accuracy, and the practical application ability of the model was studied from the perspectives of immunity, prognosis, and treatment. However, there are some limitations to the study that need to be addressed. For example, the lack of information on clinical characteristics of patients with ovarian cancer, such as TNM classification, limits our ability to include clinical characteristics in risk assessment; Second, due to the relatively small amount of data on ovarian cancer patients and the differences in data processing between the data sets, it is difficult to validate the model with broader data. The robustness of the risk scoring model needs to be further evaluated in more cohorts. Additionally, our findings require long-term *in vivo* and *in vitro* experiments to further study and verify the specific mechanisms by which acetylation modulators influence cancer development. More details about the effect of histone regulation on cancer remain to be explored.

In conclusion, based on Cox regression analysis of the expression profile of OC patients, we constructed a prognostic signature of ovarian cancer related to HAM genes, which can provide a valuable reference for identifying high-risk groups of ovarian cancer and guidance for prognostic analysis of ovarian cancer patients. Subsequently, we completed the analysis of immune infiltration, immune therapy, and mutation profiles in high- and low-risk populations. Finally, our findings may help identify more effective targeted drugs and treatment strategies for ovarian cancer patients.

## Data Availability

The datasets presented in this study can be found in online repositories. The names of the repository/repositories and accession number(s) can be found in the article/Supplementary Material.

## Supplementary material

Supplementary Table S1 List of 77 histone acetylation genes.

Supplementary Table S2 Differential expression results of 77 HAMs.

Supplementary Table S3 Results of GSEA in GO.

Supplementary Table S4 Results of GSEA in KEGG.

Supplementary Table S5 Survival time and risk grouping of patients in TCGA.

Supplementary Table S6 Summary of previous literature studies on ovarian cancer gene models.

Supplementary Table S7 RiskScore and high- and low-group assignment of samples from the IMvigor210 cohort.

Supplementary Table S8 RiskScore and high- and low-group assignment of samples from the GSE30161 cohort.

Supplementary Table S9 Results of variation analysis between high- and low-risk groups

Supplementary Figure S1 Results of LASSO regression analysis.

Supplementary Figure S2 Immune cell infiltration and clinical grading.

Supplementary Figure S3 Box plot of tumor immune dysfunction and exclusion scores in high- and low-risk groups.

Supplementary Figure S4 Kaplan–Meier survival curves of model genes.
