# Supplementary figures and images for: Establishment, immunological analysis, and drug prediction of a prognostic signature of ovarian cancer related to histone acetylation
Source: Front Pharmacol. 2022 Sep 12;13:947252. doi: 10.3389/fphar.2022.947252 (PMC9510621; doi:10.3389/fphar.2022.947252)

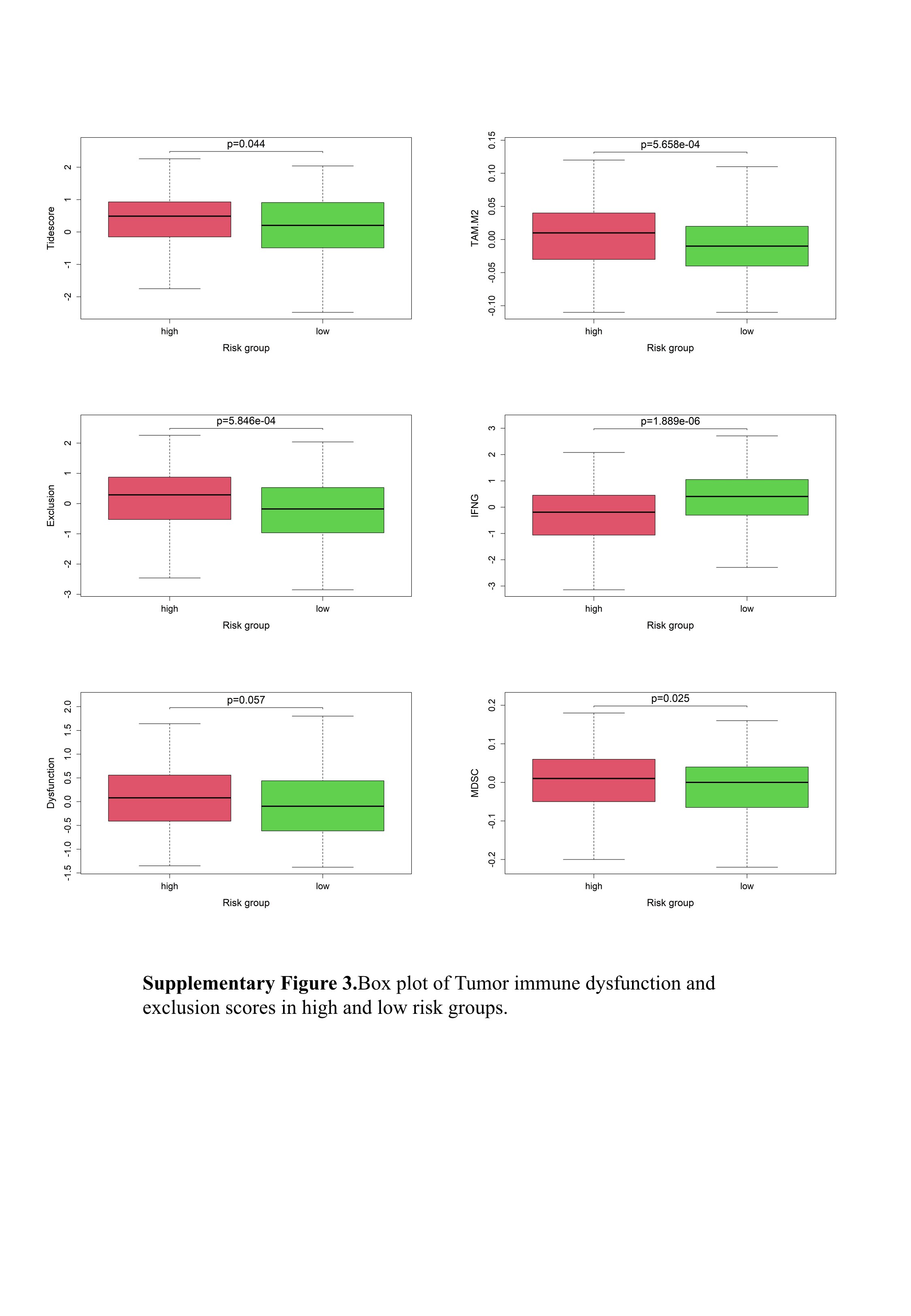

Supplement: Supplementary file 1 [file Image3.JPEG]

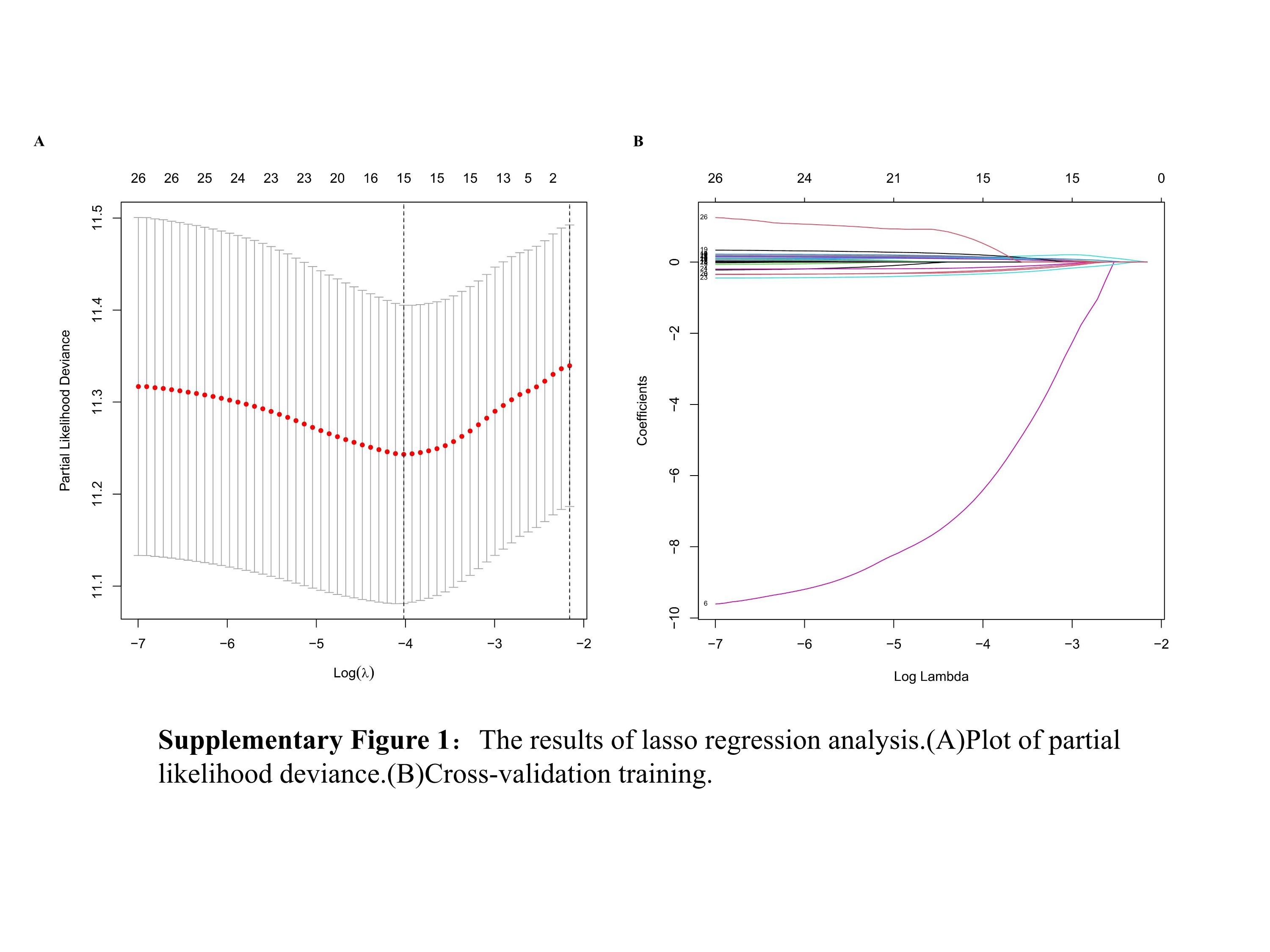

Supplement: Supplementary file 4 [file Image1.JPEG]

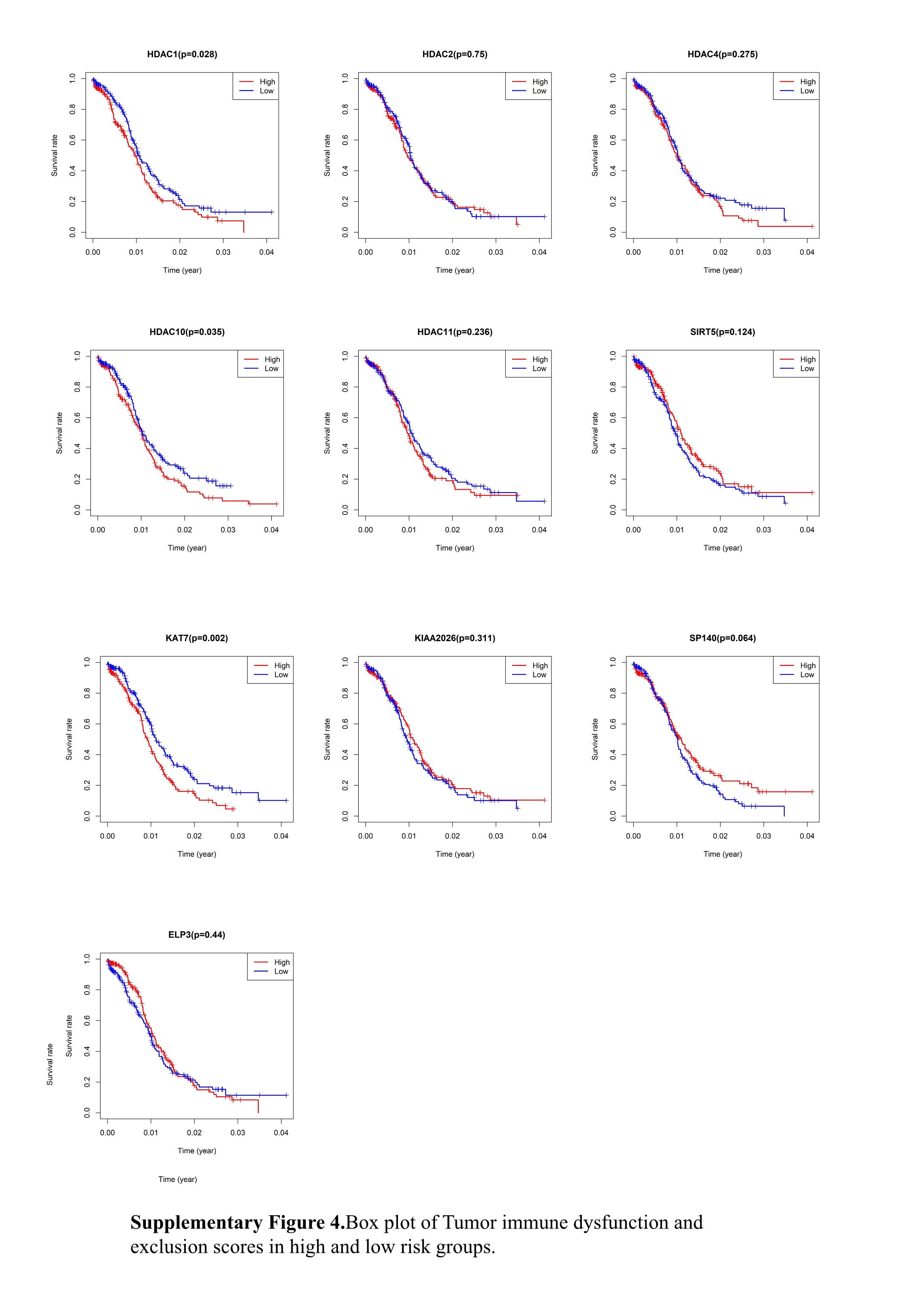

Supplement: Supplementary file 5 [file Image4.JPEG]

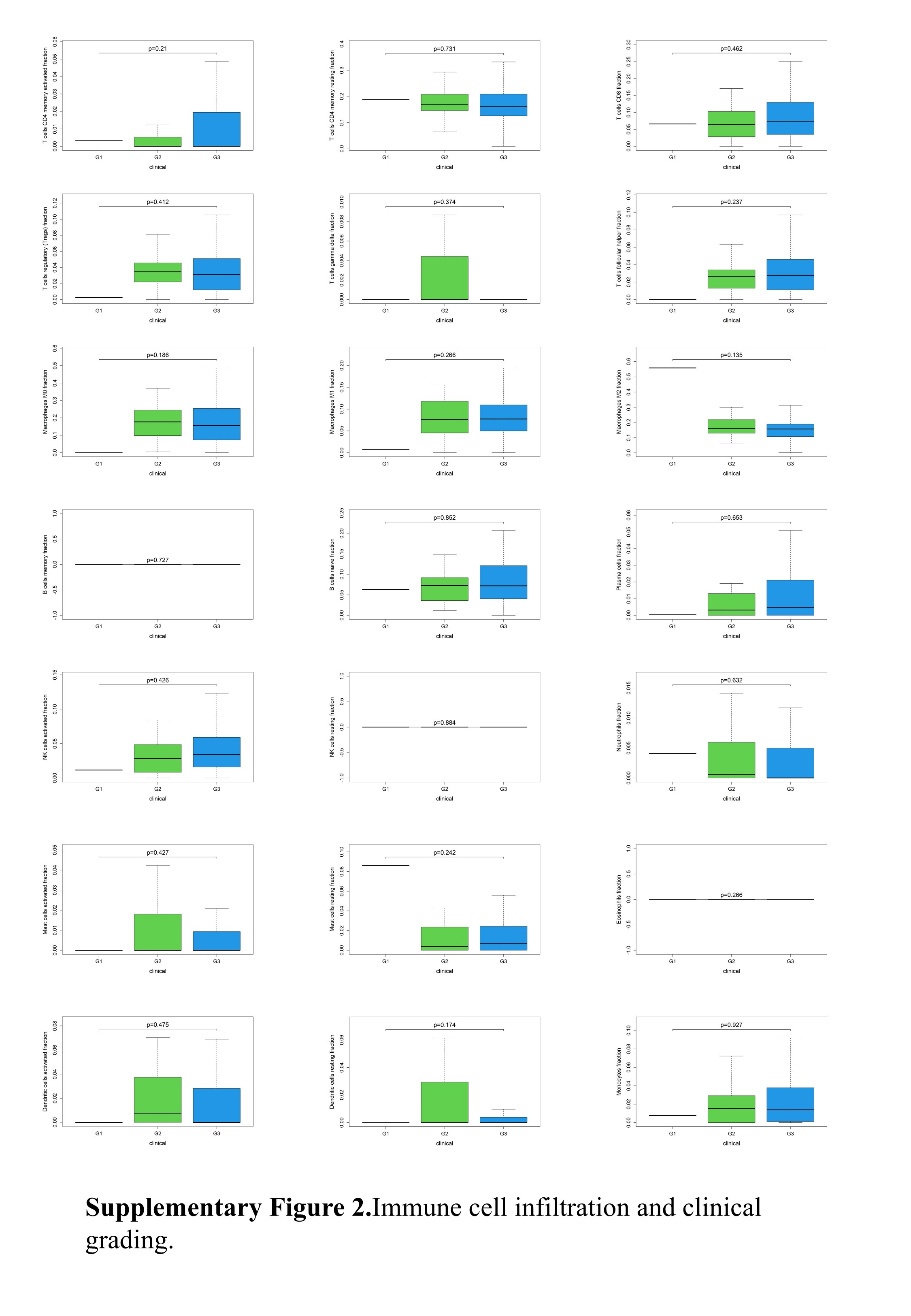

Supplement: Supplementary file 6 [file Image2.JPEG]
